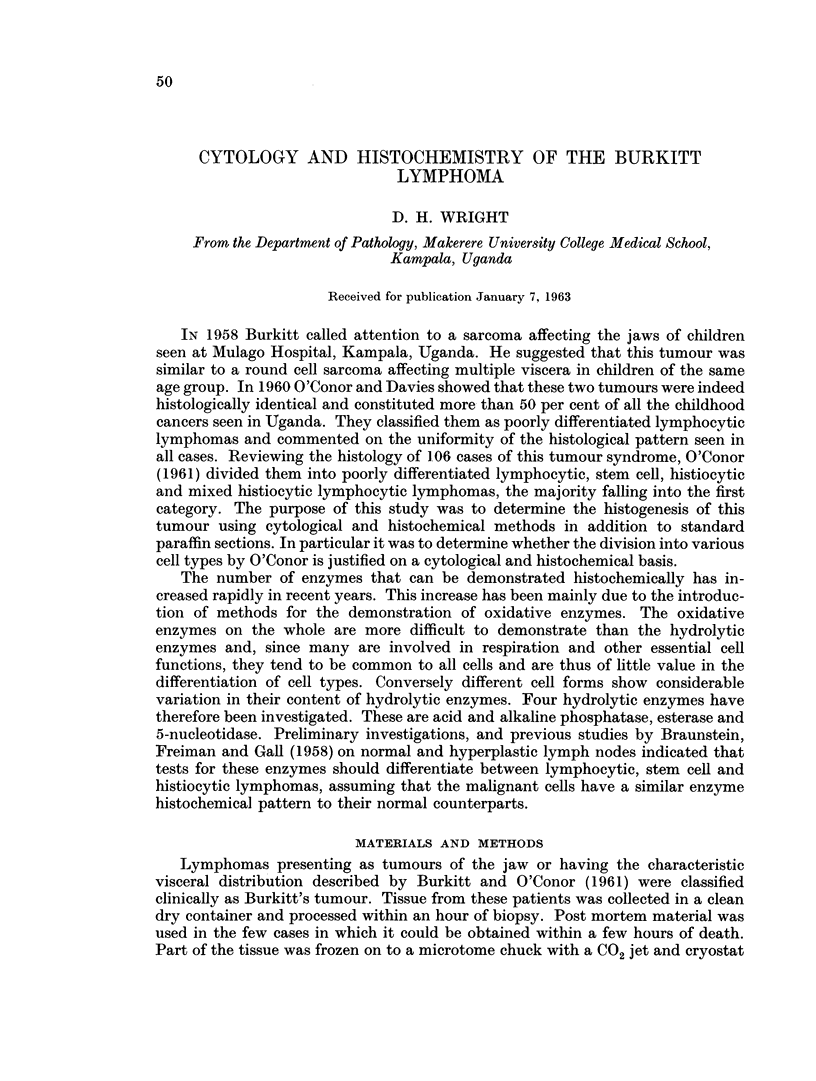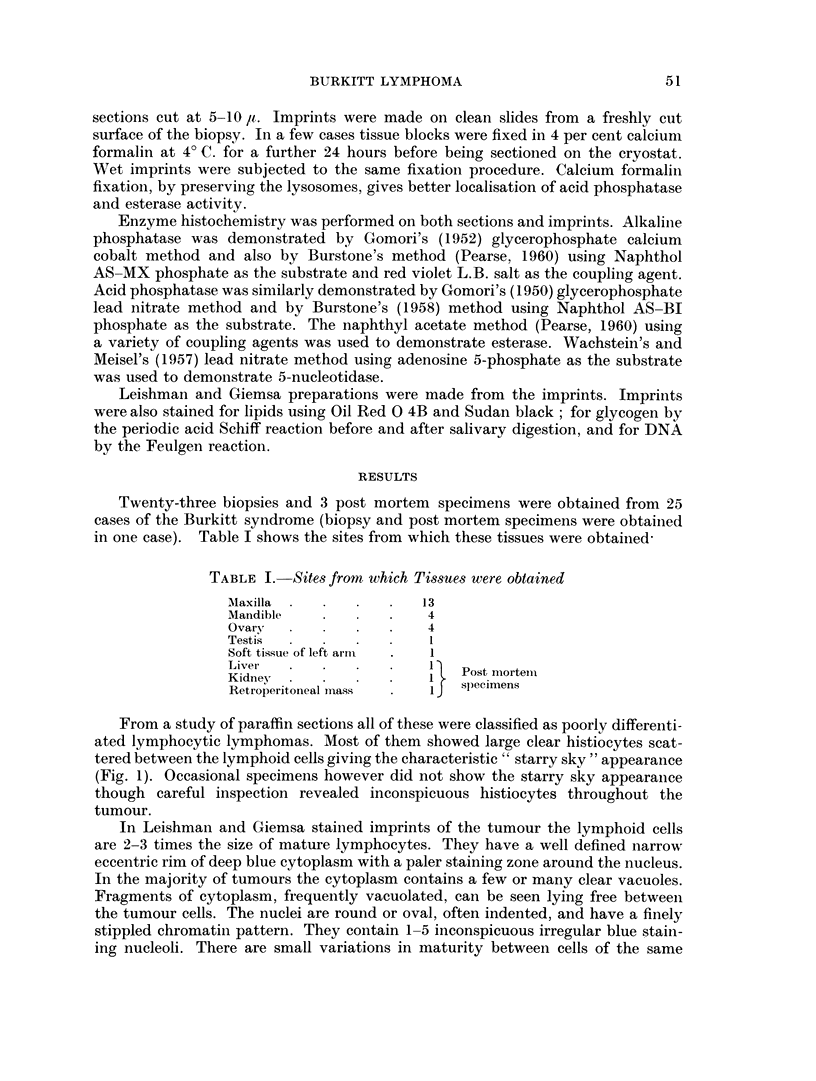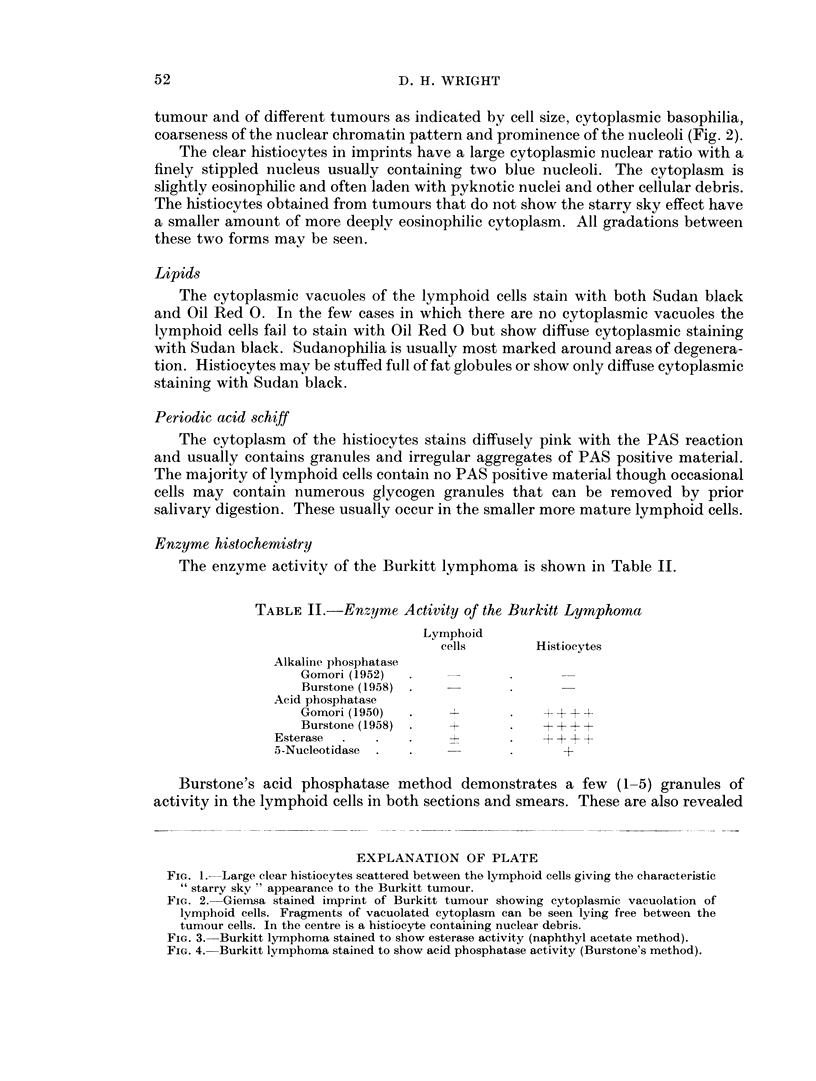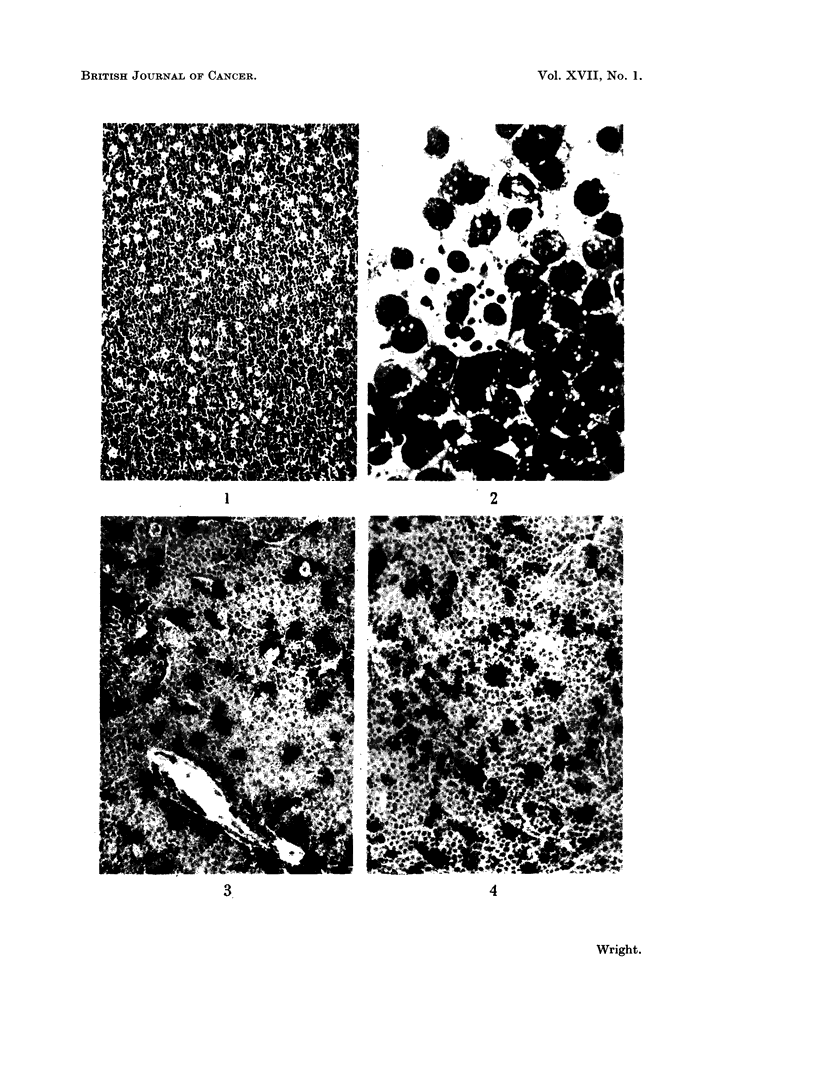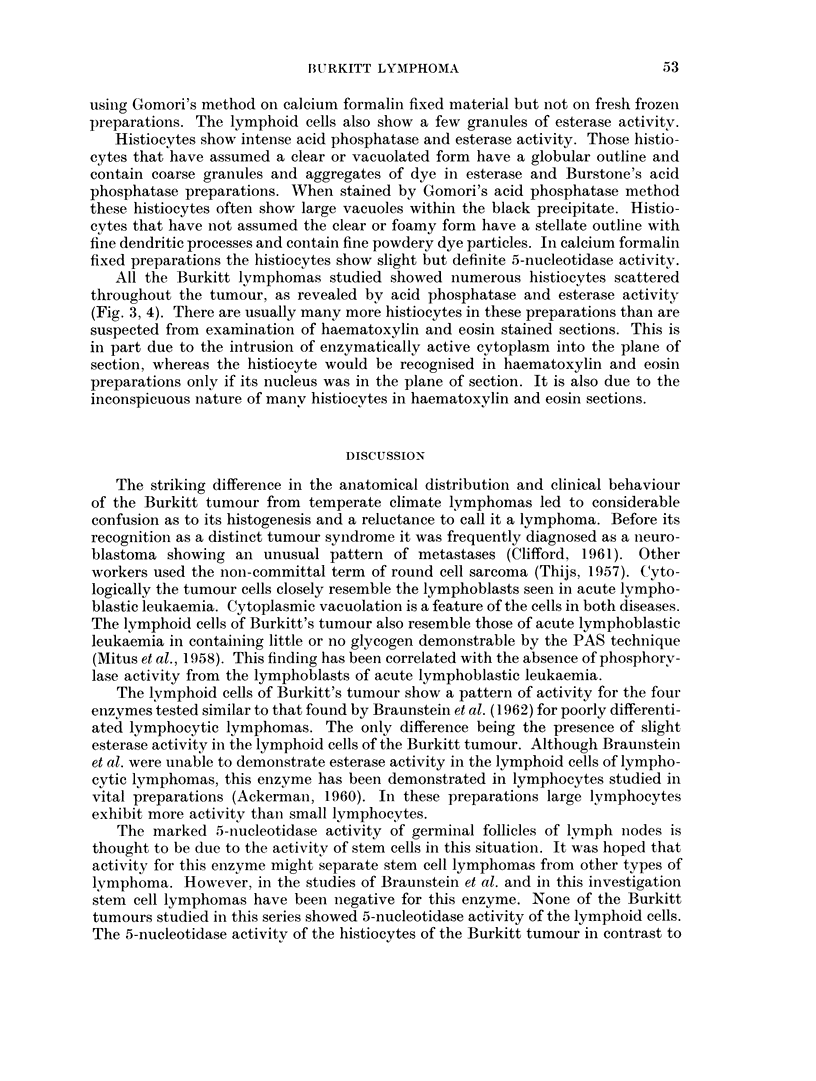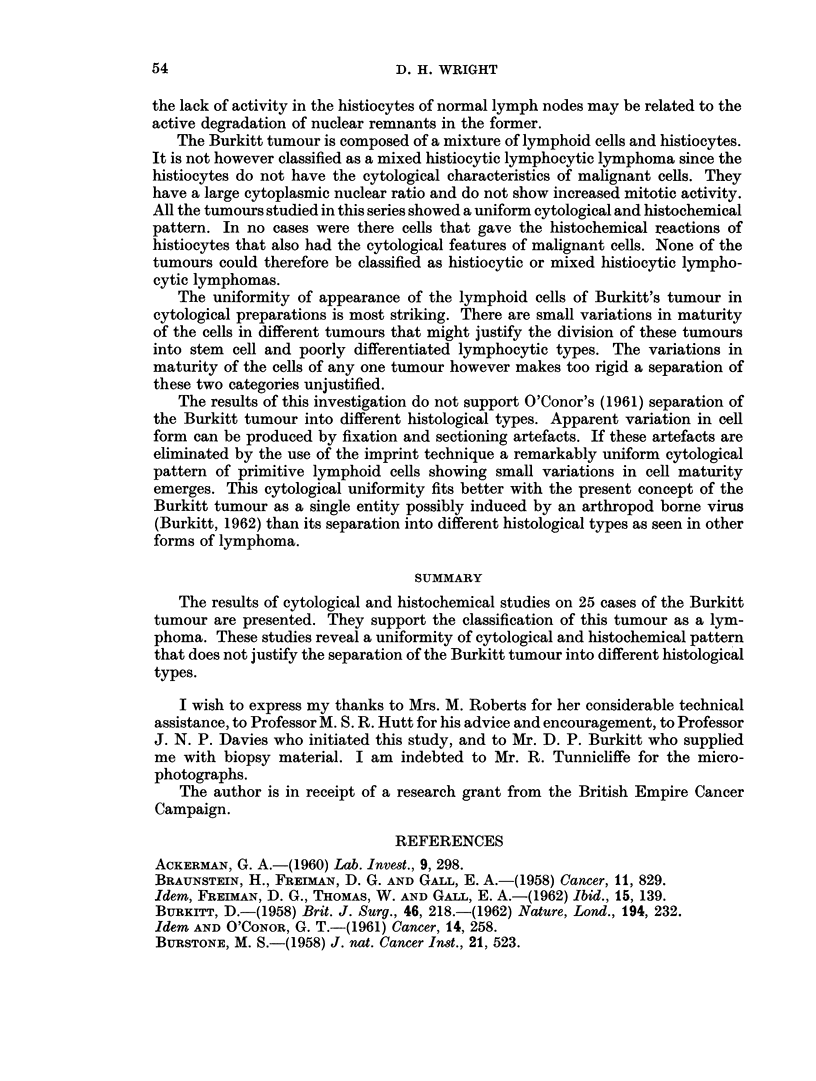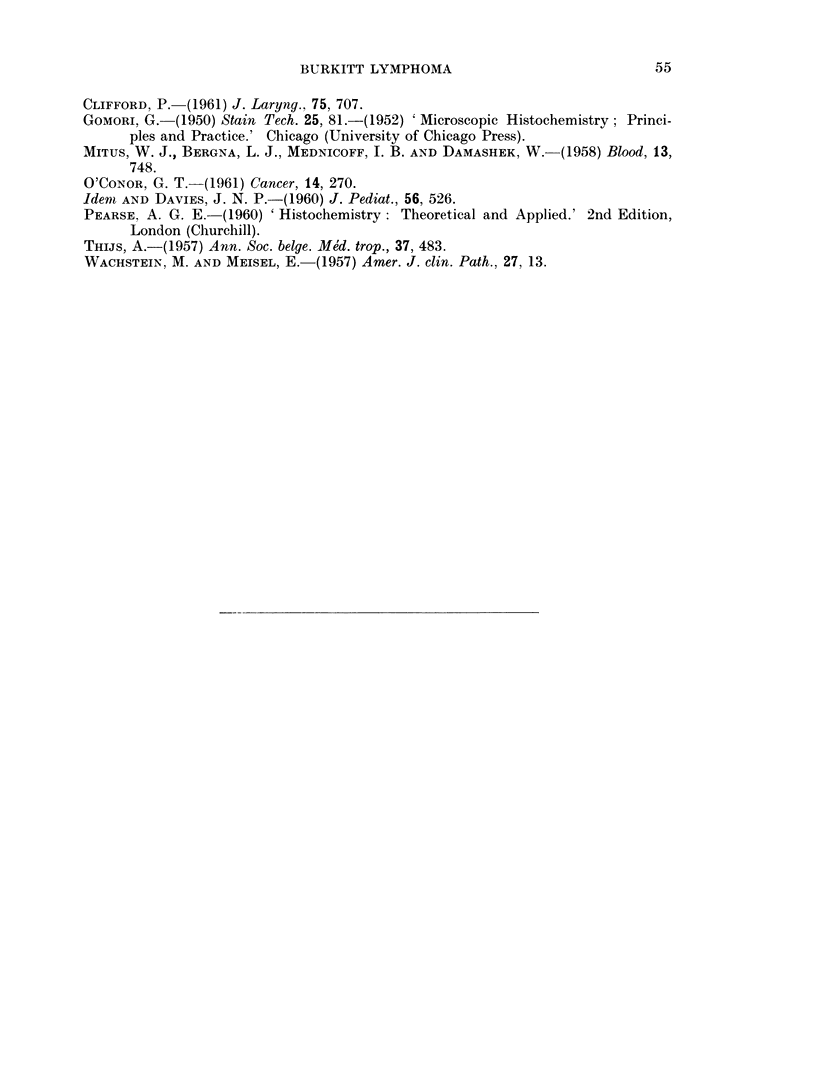# Cytology and Histochemistry of the Burkitt Lymphoma

**DOI:** 10.1038/bjc.1963.7

**Published:** 1963-03

**Authors:** D. H. Wright

## Abstract

**Images:**


### CYTOLOGY AND HISTOCHEMISTRY OF THE BURKITT LYMPHOMA

D. H. WRIGHT

*From the Department of Pathology, Makerere University College Medical School, Kampala, Uganda*

Received for publication January 7, 1963

IN 1958 Burkitt called attention to a sarcoma affecting the jaws of children seen at Mulago Hospital, Kampala, Uganda. He suggested that this tumour was similar to a round cell sarcoma affecting multiple viscera in children of the same age group. In 1960 O'Conor and Davies showed that these two tumours were indeed histologically identical and constituted more than 50 per cent of all the childhood cancers seen in Uganda. They classified them as poorly differentiated lymphocytic lymphomas and commented on the uniformity of the histological pattern seen in all cases. Reviewing the histology of 106 cases of this tumour syndrome, O'Conor (1961) divided them into poorly differentiated lymphocytic, stem cell, histiocytic and mixed histiocytic lymphocytic lymphomas, the majority falling into the first category. The purpose of this study was to determine the histogenesis of this tumour using cytological and histochemical methods in addition to standard paraffin sections. In particular it was to determine whether the division into various cell types by O'Conor is justified on a cytological and histochemical basis.

The number of enzymes that can be demonstrated histochemically has increased rapidly in recent years. This increase has been mainly due to the introduction of methods for the demonstration of oxidative enzymes. The oxidative enzymes on the whole are more difficult to demonstrate than the hydrolytic enzymes and, since many are involved in respiration and other essential cell functions, they tend to be common to all cells and are thus of little value in the differentiation of cell types. Conversely different cell forms show considerable variation in their content of hydrolytic enzymes. Four hydrolytic enzymes have therefore been investigated. These are acid and alkaline phosphatase, esterase and 5-nucleotidase. Preliminary investigations, and previous studies by Braunstein, Freiman and Gall (1958) on normal and hyperplastic lymph nodes indicated that tests for these enzymes should differentiate between lymphocytic, stem cell and histiocytic lymphomas, assuming that the malignant cells have a similar enzyme histochemical pattern to their normal counterparts.

### MATERIALS AND METHODS

Lymphomas presenting as tumours of the jaw or having the characteristic visceral distribution described by Burkitt and O'Conor (1961) were classified clinically as Burkitt's tumour. Tissue from these patients was collected in a clean dry container and processed within an hour of biopsy. Post mortem material was used in the few cases in which it could be obtained within a few hours of death. Part of the tissue was frozen on to a microtome chuck with a $CO_2$ jet and cryostat sections cut at 5–10 $\mu$. Imprints were made on clean slides from a freshly cut surface of the biopsy. In a few cases tissue blocks were fixed in 4 per cent calcium formalin at 4° C. for a further 24 hours before being sectioned on the cryostat. Wet imprints were subjected to the same fixation procedure. Calcium formalin fixation, by preserving the lysosomes, gives better localisation of acid phosphatase and esterase activity.

Enzyme histochemistry was performed on both sections and imprints. Alkaline phosphatase was demonstrated by Gomori's (1952) glycerophosphate calcium cobalt method and also by Burstone's method (Pearse, 1960) using Naphthol AS–MX phosphate as the substrate and red violet L.B. salt as the coupling agent. Acid phosphatase was similarly demonstrated by Gomori's (1950) glycerophosphate lead nitrate method and by Burstone's (1958) method using Naphthol AS–BI phosphate as the substrate. The naphthyl acetate method (Pearse, 1960) using a variety of coupling agents was used to demonstrate esterase. Wachstein's and Meisel's (1957) lead nitrate method using adenosine 5-phosphate as the substrate was used to demonstrate 5-nucleotidase.

Leishman and Giemsa preparations were made from the imprints. Imprints were also stained for lipids using Oil Red O 4B and Sudan black ; for glycogen by the periodic acid Schiff reaction before and after salivary digestion, and for DNA by the Feulgen reaction.

### RESULTS

Twenty-three biopsies and 3 post mortem specimens were obtained from 25 cases of the Burkitt syndrome (biopsy and post mortem specimens were obtained in one case). Table I shows the sites from which these tissues were obtained·

TABLE I.—*Sites from which Tissues were obtained*

| | | |
|---|---|---|
| Maxilla | 13 | |
| Mandible | 4 | |
| Ovary | 4 | |
| Testis | 1 | |
| Soft tissue of left arm | 1 | |
| Liver | 1 | Post mortem specimens |
| Kidney | 1 | |
| Retroperitoneal mass | 1 | |

From a study of paraffin sections all of these were classified as poorly differentiated lymphocytic lymphomas. Most of them showed large clear histiocytes scattered between the lymphoid cells giving the characteristic " starry sky " appearance (Fig. 1). Occasional specimens however did not show the starry sky appearance though careful inspection revealed inconspicuous histiocytes throughout the tumour.

In Leishman and Giemsa stained imprints of the tumour the lymphoid cells are 2–3 times the size of mature lymphocytes. They have a well defined narrow eccentric rim of deep blue cytoplasm with a paler staining zone around the nucleus. In the majority of tumours the cytoplasm contains a few or many clear vacuoles. Fragments of cytoplasm, frequently vacuolated, can be seen lying free between the tumour cells. The nuclei are round or oval, often indented, and have a finely stippled chromatin pattern. They contain 1–5 inconspicuous irregular blue staining nucleoli. There are small variations in maturity between cells of the same tumour and of different tumours as indicated by cell size, cytoplasmic basophilia, coarseness of the nuclear chromatin pattern and prominence of the nucleoli (Fig. 2).

The clear histiocytes in imprints have a large cytoplasmic nuclear ratio with a finely stippled nucleus usually containing two blue nucleoli. The cytoplasm is slightly eosinophilic and often laden with pyknotic nuclei and other cellular debris. The histiocytes obtained from tumours that do not show the starry sky effect have a smaller amount of more deeply eosinophilic cytoplasm. All gradations between these two forms may be seen.

*Lipids*

The cytoplasmic vacuoles of the lymphoid cells stain with both Sudan black and Oil Red O. In the few cases in which there are no cytoplasmic vacuoles the lymphoid cells fail to stain with Oil Red O but show diffuse cytoplasmic staining with Sudan black. Sudanophilia is usually most marked around areas of degeneration. Histiocytes may be stuffed full of fat globules or show only diffuse cytoplasmic staining with Sudan black.

*Periodic acid schiff*

The cytoplasm of the histiocytes stains diffusely pink with the PAS reaction and usually contains granules and irregular aggregates of PAS positive material. The majority of lymphoid cells contain no PAS positive material though occasional cells may contain numerous glycogen granules that can be removed by prior salivary digestion. These usually occur in the smaller more mature lymphoid cells.

*Enzyme histochemistry*

The enzyme activity of the Burkitt lymphoma is shown in Table II.

TABLE II.—*Enzyme Activity of the Burkitt Lymphoma*

| | Lymphoid cells | Histiocytes |
|---|---|---|
| Alkaline phosphatase | | |
| Gomori (1952) | — | — |
| Burstone (1958) | — | — |
| Acid phosphatase | | |
| Gomori (1950) | + | ++++ |
| Burstone (1958) | + | ++++ |
| Esterase | ± | ++++ |
| 5-Nucleotidase | — | + |

Burstone's acid phosphatase method demonstrates a few (1–5) granules of activity in the lymphoid cells in both sections and smears. These are also revealed

EXPLANATION OF PLATE

FIG. 1.—Large clear histiocytes scattered between the lymphoid cells giving the characteristic " starry sky " appearance to the Burkitt tumour.

FIG. 2.—Giemsa stained imprint of Burkitt tumour showing cytoplasmic vacuolation of lymphoid cells. Fragments of vacuolated cytoplasm can be seen lying free between the tumour cells. In the centre is a histiocyte containing nuclear debris.

FIG. 3.—Burkitt lymphoma stained to show esterase activity (naphthyl acetate method).

FIG. 4.—Burkitt lymphoma stained to show acid phosphatase activity (Burstone's method).

1 2 3 4

Wright.

using Gomori's method on calcium formalin fixed material but not on fresh frozen preparations. The lymphoid cells also show a few granules of esterase activity.

Histiocytes show intense acid phosphatase and esterase activity. Those histiocytes that have assumed a clear or vacuolated form have a globular outline and contain coarse granules and aggregates of dye in esterase and Burstone's acid phosphatase preparations. When stained by Gomori's acid phosphatase method these histiocytes often show large vacuoles within the black precipitate. Histiocytes that have not assumed the clear or foamy form have a stellate outline with fine dendritic processes and contain fine powdery dye particles. In calcium formalin fixed preparations the histiocytes show slight but definite 5-nucleotidase activity.

All the Burkitt lymphomas studied showed numerous histiocytes scattered throughout the tumour, as revealed by acid phosphatase and esterase activity (Fig. 3, 4). There are usually many more histiocytes in these preparations than are suspected from examination of haematoxylin and eosin stained sections. This is in part due to the intrusion of enzymatically active cytoplasm into the plane of section, whereas the histiocyte would be recognised in haematoxylin and eosin preparations only if its nucleus was in the plane of section. It is also due to the inconspicuous nature of many histiocytes in haematoxylin and eosin sections.

### DISCUSSION

The striking difference in the anatomical distribution and clinical behaviour of the Burkitt tumour from temperate climate lymphomas led to considerable confusion as to its histogenesis and a reluctance to call it a lymphoma. Before its recognition as a distinct tumour syndrome it was frequently diagnosed as a neuroblastoma showing an unusual pattern of metastases (Clifford, 1961). Other workers used the non-committal term of round cell sarcoma (Thijs, 1957). Cytologically the tumour cells closely resemble the lymphoblasts seen in acute lymphoblastic leukaemia. Cytoplasmic vacuolation is a feature of the cells in both diseases. The lymphoid cells of Burkitt's tumour also resemble those of acute lymphoblastic leukaemia in containing little or no glycogen demonstrable by the PAS technique (Mitus *et al.*, 1958). This finding has been correlated with the absence of phosphorylase activity from the lymphoblasts of acute lymphoblastic leukaemia.

The lymphoid cells of Burkitt's tumour show a pattern of activity for the four enzymes tested similar to that found by Braunstein *et al.* (1962) for poorly differentiated lymphocytic lymphomas. The only difference being the presence of slight esterase activity in the lymphoid cells of the Burkitt tumour. Although Braunstein *et al.* were unable to demonstrate esterase activity in the lymphoid cells of lymphocytic lymphomas, this enzyme has been demonstrated in lymphocytes studied in vital preparations (Ackerman, 1960). In these preparations large lymphocytes exhibit more activity than small lymphocytes.

The marked 5-nucleotidase activity of germinal follicles of lymph nodes is thought to be due to the activity of stem cells in this situation. It was hoped that activity for this enzyme might separate stem cell lymphomas from other types of lymphoma. However, in the studies of Braunstein *et al.* and in this investigation stem cell lymphomas have been negative for this enzyme. None of the Burkitt tumours studied in this series showed 5-nucleotidase activity of the lymphoid cells. The 5-nucleotidase activity of the histiocytes of the Burkitt tumour in contrast to the lack of activity in the histiocytes of normal lymph nodes may be related to the active degradation of nuclear remnants in the former.

The Burkitt tumour is composed of a mixture of lymphoid cells and histiocytes. It is not however classified as a mixed histiocytic lymphocytic lymphoma since the histiocytes do not have the cytological characteristics of malignant cells. They have a large cytoplasmic nuclear ratio and do not show increased mitotic activity. All the tumours studied in this series showed a uniform cytological and histochemical pattern. In no cases were there cells that gave the histochemical reactions of histiocytes that also had the cytological features of malignant cells. None of the tumours could therefore be classified as histiocytic or mixed histiocytic lymphocytic lymphomas.

The uniformity of appearance of the lymphoid cells of Burkitt's tumour in cytological preparations is most striking. There are small variations in maturity of the cells in different tumours that might justify the division of these tumours into stem cell and poorly differentiated lymphocytic types. The variations in maturity of the cells of any one tumour however makes too rigid a separation of these two categories unjustified.

The results of this investigation do not support O'Conor's (1961) separation of the Burkitt tumour into different histological types. Apparent variation in cell form can be produced by fixation and sectioning artefacts. If these artefacts are eliminated by the use of the imprint technique a remarkably uniform cytological pattern of primitive lymphoid cells showing small variations in cell maturity emerges. This cytological uniformity fits better with the present concept of the Burkitt tumour as a single entity possibly induced by an arthropod borne virus (Burkitt, 1962) than its separation into different histological types as seen in other forms of lymphoma.

### SUMMARY

The results of cytological and histochemical studies on 25 cases of the Burkitt tumour are presented. They support the classification of this tumour as a lymphoma. These studies reveal a uniformity of cytological and histochemical pattern that does not justify the separation of the Burkitt tumour into different histological types.

I wish to express my thanks to Mrs. M. Roberts for her considerable technical assistance, to Professor M. S. R. Hutt for his advice and encouragement, to Professor J. N. P. Davies who initiated this study, and to Mr. D. P. Burkitt who supplied me with biopsy material. I am indebted to Mr. R. Tunnicliffe for the microphotographs.

The author is in receipt of a research grant from the British Empire Cancer Campaign.

### REFERENCES

Ackerman, G. A.—(1960) *Lab. Invest.*, **9**, 298.
Braunstein, H., Freiman, D. G. and Gall, E. A.—(1958) *Cancer*, **11**, 829.
*Idem*, Freiman, D. G., Thomas, W. and Gall, E. A.—(1962) *Ibid.*, **15**, 139.
Burkitt, D.—(1958) *Brit. J. Surg.*, **46**, 218.—(1962) *Nature, Lond.*, **194**, 232.
*Idem* and O'Conor, G. T.—(1961) *Cancer*, **14**, 258.
Burstone, M. S.—(1958) *J. nat. Cancer Inst.*, **21**, 523.

CLIFFORD, P.—(1961) *J. Laryng.*, **75**, 707.
GOMORI, G.—(1950) *Stain Tech.* **25**, 81.—(1952) 'Microscopic Histochemistry; Principles and Practice.' Chicago (University of Chicago Press).
MITUS, W. J., BERGNA, L. J., MEDNICOFF, I. B. AND DAMASHEK, W.—(1958) *Blood*, **13**, 748.
O'CONOR, G. T.—(1961) *Cancer*, **14**, 270.
*Idem* AND DAVIES, J. N. P.—(1960) *J. Pediat.*, **56**, 526.
PEARSE, A. G. E.—(1960) 'Histochemistry: Theoretical and Applied.' 2nd Edition, London (Churchill).
THIJS, A.—(1957) *Ann. Soc. belge. Méd. trop.*, **37**, 483.
WACHSTEIN, M. AND MEISEL, E.—(1957) *Amer. J. clin. Path.*, **27**, 13.